# Parathyroid Hormone-Related Peptide (PTHrP): Evaluation of Pediatric, Covariate-Stratified Reference Intervals

**DOI:** 10.3390/children9060896

**Published:** 2022-06-15

**Authors:** Vincenzo Brescia, Antonietta Fontana, Roberto Lovero, Carmela Capobianco, Stella Vita Marsico, Tiziana De Chirico, Carla Pinto, Elisa Mascolo, Angela Pia Cazzolla, Maria Felicia Faienza, Francesca Di Serio

**Affiliations:** 1 Clinical Pathology Unit, AOU Policlinico Consorziale di Bari-Ospedale Giovanni XXIII, 70124 Bari, Italy; bresciavincenzo59@gmail.com (V.B.); a-fontana@libero.it (A.F.); carmenbiac@yahoo.it (C.C.); mastella64@libero.it (S.V.M.); tizianadechirico@inwind.it (T.D.C.); carlapinto156@gmail.com (C.P.); elisa.mascolo@libero.it (E.M.); francesca.diserio@policlinico.ba.it (F.D.S.); 2 Department of Clinical and Experimental Medicine, Università degli Studi di Foggia, 71122 Foggia, Italy; elicio@inwind.it; 3 Pediatric Unit, Department of Biomedical Sciences and Human Oncology, University of Bari “Aldo Moro”, 70121 Bari, Italy; mariafelicia.faienza@uniba.it

**Keywords:** parathyroid hormones related protein, hypercalcemia, reference range

## Abstract

Parathyroid hormone-related peptide (PTHrP) is expressed at a wide range of sites in the body and performs different functions including vasodilation, relaxation of smooth muscle cells, and regulation of bone development. PTHrP also mediates hypercalcemia related to neoplastic diseases. However, reference ranges specific method and age were not evaluated. We establish PTHrP reference ranges in apparently healthy, normocalcemic, normophosphatemic pediatric individuals. In this observational prospective, study we measured PTHrP in serum from 178 samples (55.06% male 44.94% female) from apparently healthy pediatric subjects [median age 10 years (range 1–18)] subunit ELISA method The statistical analysis performed provided for the calculation of the 95% reference interval, right-sided, with a non-parametric percentile method (CLSI C28-A3). Upper reference limits (URL) for PTHrP was 2.89 ng/mL (2.60 to 3.18; 90% CI). No significant differences were found between the median PTHrP concentrations in males vs females and in the age range categories selected. Comprehensive normal values for PTHrP are indispensable to the assessment of calcium phosphorus dysfunction in children. Severe hypercalcemia is a rare, but clinically significant condition, in infancy and childhood. PTHrP values higher than the reference value may help to distinguish the hypercalcemic product of a malignancy, paraneoplastic syndromes mediated by PTHrP, from other causes.

## 1. Introduction

Parathyroid hormone related protein (PTHrP) is a multifaceted protein with several biologically active domains that regulate its many roles in normal physiology and human disease. PTHrP has several isoforms, ranging in size from 60 to 173 amino acids, created by differential splicing and post-translational processing by prohormonal convertases [1,2]. The gene, PTHHL, which codes for PTHrP, is located on chromosome 12 and has nine exons. PTHrP has multiple domains, each with different biological functions [3]. The first 36 amino acids (1–36) encode a domain that controls the intracellular trafficking of PTHrP precursors before being cleaved when the mature molecule is secreted. Domain (1–34) allows PTHrP to bind to and activate the PTH type 1 receptor (PTH1R), a “G protein receptor”. Eight of the first 13 residues within this region (1–34) of PTHrP are identical to PTH [4]. Although PTH and PTHrP are structurally and sometimes functionally similar, they exert different effects due to differences in targets and structure-function relationships [5]. The amino acid sequence 67–94 is important for the intracrine actions of PTHrP, including the regulation of cell proliferation, survival and apoptosis [6].

The domain starting from residue 107 is associated with inhibition of osteoclast-mediated bone resorption and anabolic effects on bone (“osteostatin” region) [7]. PTHrP can play different roles in mitogenesis depending on the way the signal is activated. PTHrP by binding to PTH1R inhibits cell proliferation (paracrine/autocrine), in addition PTHrP can translocate into the nucleus and paradoxically increase proliferation (intracrine actions) [8,9].

PTHrP is highly expressed in human tissues and plays a role in many physiological processes such as mammary gland development, tooth eruption, keratinocyte differentiation for hair follicle development, chondrocyte maturation and bone formation endochondral (autocrine and paracrine action) [3].

PTHrP intervenes in the physiological regulation of bone remodeling. It is produced locally (paracrine function) by the progenitors of osteoblasts; acts by promoting the differentiation of mature osteoblasts by inhibiting the apoptosis process that allows bone formation; it also stimulates the differentiation of osteoclasts responsible for bone resorption instead [10].

PTHrP intervenes in the transepithelial transport of calcium, in particular in the kidney and in the mammary gland; in the relaxation of the smooth muscles of the uterus, bladder, gastrointestinal tract and arterial wall; acts on cell proliferation, differentiation and apoptosis of multiple tissues. Moreover, it is an indispensable hormone for the evolution of pregnancy and for fetal development [3]. Unlike PTH, it acts in many tissues as a paracrine or autocrine factor rather than a classical hormone [3,10]. In the kidney, PTHrP is abundantly expressed and upregulated, showing growth-modulatory and pro-inflammatory properties [11].

PTHrP is synthesized and released from various normal tissues but also from neoplastic tissues [5].

Hypercalcemia of malignant tumors in children is a complication that occurs in 0.4–1.3% of cancer cases, it is a severe condition with a risk of fatal outcomes due to cardiac or neurological complications [12].

PTHrP produced by malignant tumors is responsible for severe hypercalcemia (HHM), a rare but clinically significant condition in infancy and childhood. Benign tumors, sarcomas and hematolymphatic neoplasms can also produce PTHrP [13,14,15,16,17,18,19]. The hypercalcemia associated with pheochromocytoma may be due to secretion of PTHrP [20].

The paraneoplastic syndrome humoral hypercalcemia of malignancy (H.H.M.) leads to increased osteoclastic bone resorption and serum calcium levels [16] produces effects similar to those of primary hyperparathyroidism therefore a careful and correct request and interpretation of laboratory tests is necessary for the differential diagnosis between the two pathological conditions [21]. In HHM, typical laboratory values include elevated Ca and PTHrP levels [22].

However, the methods for the assay of PTHrP are often not sufficiently standardized. In particular, there are no accurate decision limits and reference values selected on the basis of the method and covariables (age, sex) [23].

The aim of this study is to determine accurate reference ranges for PTHrP in apparently healthy pediatric individuals. Serum PTHrP was measured with an immunometric method (ELISA).

## 2. Materials and Methods

### 2.1. Study Design and Participants

Before the start of the study, ethical approval was obtained from the relevant institutional review committees of the Policlinico University Hospital of Bari (Biomarkers of Bone Metabolism; Study number. 38359/COMET of 27 April 2021 BMOPed). The prospective observational study was conducted in accordance with the principles of the Declaration of Helsinki and the International Conference on Harmonization Guidelines for Good Clinical Practice.

Apparently healthy individuals between the ages of 1 and 18 were enrolled. The key inclusion criteria were: serum calcium range 8.5–10.6 mg/dL, serum phosphate range 3.1–5.90 mg/dL and serum creatinine range 0.6–1.17 mg/dL. The key exclusion criteria were pregnancy, known endocrine/metabolic diseases; hypocalcemia, hypophosphatemia, hypercalcemia, hyperphostatemia evaluated according to the indicated acceptability ranges; renal insufficiency (eGFR < 75 mL/min/1.73 m^2^), 25 (OH) vitamin D overdose/intoxication (>250 nmol/L (>100 ng/mL)); cancer; hospitalization in the last 4 months; bone fracture (≤3 months). Taking hormone replacement therapy; 25 (OH) vitamin D deficiency status based on circulating levels <50 nmol/L (<20 ng/mL).

### 2.2. Sample Management

Blood samples were collected for one year (January 2021 and February 2022). During the entire collection period, the individual subject was selected only once to provide a sample. Blood from the forearm vein was collected into 5-mL Vacutainer tubes with no anticoagulant and for the standardization of the collection procedures all of the samples were collected in the time 8.00–10.00 am. The blood samples were centrifuged (1000× *g*, 15 min, 4 °C). Aliquots of serum samples were prepared within 2 h of venipuncture and immediately frozen and stored at −70 °C until determination in the laboratory (Clinical Pathology; University Hospital Policlinico di Bari).

Serum PTHrP was measured with “competitive” enzyme immunoassay (ELISA) designed to measure subunit (1–34) (Parathyroid hormone related protein (PTHrP) (1–34) Kit EIA, (Catalog No. EK-056-04) (PHOENIX PHARMACEUTICALS, INC., 330 Beach Rd., Burlingame, CA 94010, USA)).

The method is based on competition between biotinylated PTHrP (1–34) and target PTHrP (1–34) present in the standard peptide solution or serum sample for binding to the primary antibody. A standard curve can be established by plotting the measured optical density as a function of various known standard concentrations of PTHrP (1–34). The concentration of PTHrP (1–34) in the samples can then be determined by extrapolation of the data. The method has an analytical sensitivity of 0.15 ng/mL, a linear range of 0.5–4.46 ng/mL, an intra-assay variation <10% and inter-assay variation <15%. The test does not cross-react with PTH. The dosage of PTHrP was performed with ELISA assay using the DSX^®^ TGSTA Dynex Technologies, Inc. (Chantilly, VA, USA) respecting the manufacturer’s instructions and carrying out suitable internal quality controls.

### 2.3. Analytical Determination

The dosage of 25 (OH) Vitamin D was performed with chemiluminescence tests using the TGSTA Technogenetics instrumentation (Technogenetics, Milan, Italy)

The dosage of serum calcium (v.n. 8.5–10.6 mg/dL), phosphorus (v.n. 3.1–5.90 mg/dL), was carried out using the colorimetric method, creatinine (v.n. 0.6–1.17 mg/dL), was carried out by enzyme method on Dimension VISTA 1500 instrumentation (Siemens, Munich, Germany).

### 2.4. Statistical Analyzes

We used a posteriori approach, as exclusion criteria were applied after samples were collected from all respondents

The PTHrP reference intervals [median 5–95 percentile and 90% confidence intervals (CI) of the upper reference limits (URLs)] were calculated using standard non-parametric statistical analyses, according to CLSI EP28-A3c [24]. Scatterplots were created to visually inspect the data; we removed outliers in partitions with normally distributed data using the Tukey test [25,26]. Only an upper limit of normality was used as only high values are suspicious.

The measurements are modelled on age using weighted polynomial regression (Altman and Chitty, 1994). This regression model gives the mean of the measurements as a function of age: mean (age). A scatter plot was used to visualize the distribution of the measurements versus age with evidence of the calculated mean (central line) and centile curves. The “age-related reference interval” was calculated for the stratification of subgroups in order to verify any variations in the patients’ age.

Mann-Whitney U test has been used to evaluate whether PTHrP levels stratified by sex and age were from the same population.

To calculate the significance of the differences, the *p*-value was calculated. A *p*-value < 0.05 was considered statistically significant. The MedCalc^®^ (11.6.1.0) program was used for statistical analysis.

## 3. Results

A total of 184 samples were collected and 178 samples were deemed evaluable; Before the statistical evaluation, n. 4 (2.17%) PTHrP values from the statistical analysis as they had insufficient vitamin D concentrations for the pediatric age; n. 2 (1.08%) PTHrP values were considered suspected outliers by applying Tukey’s statistical criterion. In the group of subjects evaluated, 98 (55.06%) individuals were male and 80 (44.94%) were female. The statistical evaluation of the PTHrP values obtained in all subjects and in the two groups stratified by sex (male: PTHRP-M; female PTHrP-F) is reported in Table 1.

**Figure 1:**
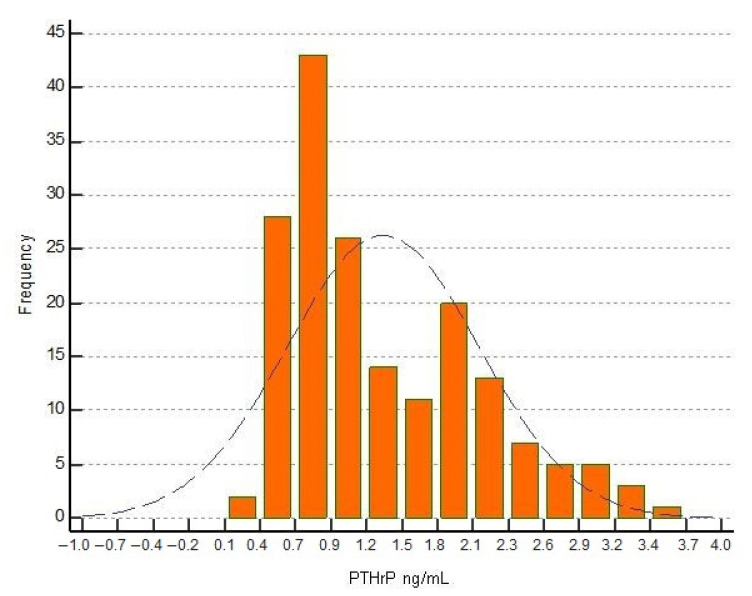
Frequency (%) histogram of pediatric PTHrP (ng/mL) concentrations evaluated.

**Figure 2:**
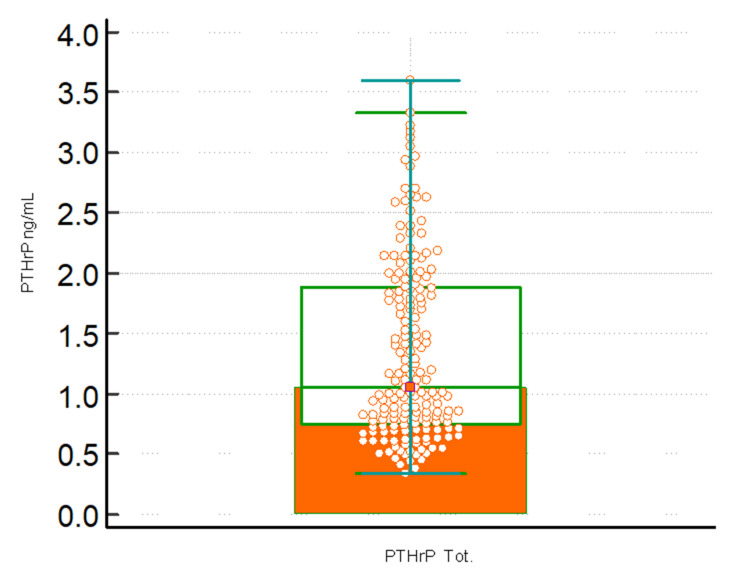
Boxplot with the statistical summary of the concentration of PTHrP (ng/mL) obtained on the total of the subjects evaluated (PTHrP Tot.). The central box shows the values from the 25th to the 75th quartile, the central line the median, the horizontal lines the extension from the minimum to the maximum value (range). A possible outside is highlighted.

Across all samples, the lowest and highest PTHrP serum were 0.34 ng/mL and 3.66 ng/mL, respectively; the median concentration was 1.08 ng/mL (0.50 to 2.81 (5.0–95.0th percentile) ng/mL). Upper limit PTHrP concentrations obtained on all samples calculated as 95% reference interval, right-sided, non-parametric percentile method (according to CLSI C28-A3), was 2.89 ng/mL (2.60 to 3.18; 90% CI). 43% of the samples had a concentration between 0.7 and 0.9 ng/mL. The data are presented in Figure 1 showing the frequency histogram as a function of the PTHrP concentration. Figure 2 displays the boxplot for viewing the statistical summary of the concentration of PTHrP obtained on the total of the subjects evaluated.

## 4. Categorical Analysis

The difference in the distribution by sex (Males 55.06%) is within the pre-established acceptability criteria (protocol-specified: 50 ± 5%). Stratification by sex showed a median of 1.00 ng/mL (0.45 to 2.78; 5–95 percentile) in males (PTHrP-M); a median of 1.20 ng/mL (0.53 to 2.83; (5.0–95.0 percentile) ng/mL) in females (PTHrP-F). The analysis of the reference intervals stratified by age (2, 4, 6, 8, 10, 12, 14, 16, 18 years) reported in Table 2, highlighted the median value of the highest and lowest PTHrP respectively equal to 3.98 ng/mL (95.0 percentile) in the two-year age group and 2.23 ng/mL (95.0 percentile) in the 6-year age group.

**Figure 3:**
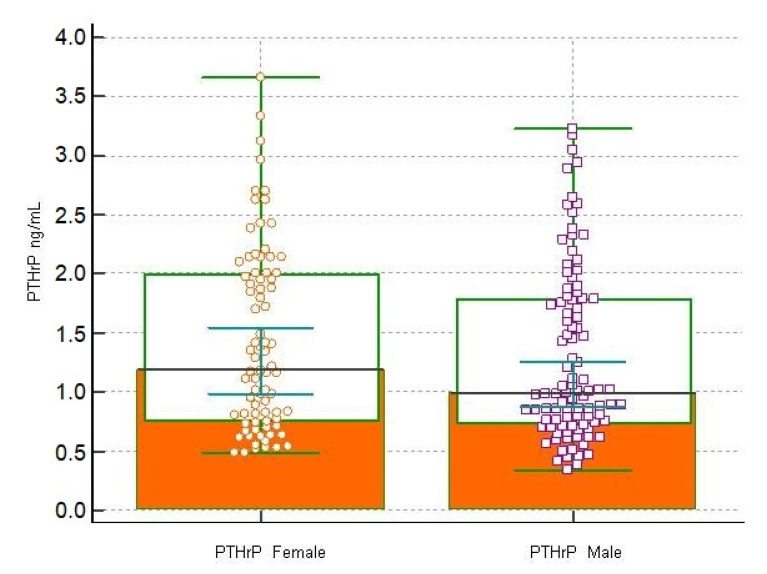
Plot of the scatter diagram of the measurements versus sex (Female and Male). The central box shows the values from the 25th to the 75th quartile, the central line the median, the horizontal lines the extension from the minimum to the maximum value (range).

The plot of the scatter diagram of the measurements versus age with the calculated mean (central line) and centile curves allowed to highlight three groups of subjects with apparent different distribution of values: (1) 1–5 years, (2) 6–12 years and (3) 13–18 years (Figure 3). These age limits were used to evaluate any changes in the concentration of PTHrP to be related to age. The statistical analysis showing the concentrations evaluated according to age is shown in Table 3.

**Figure 4:**
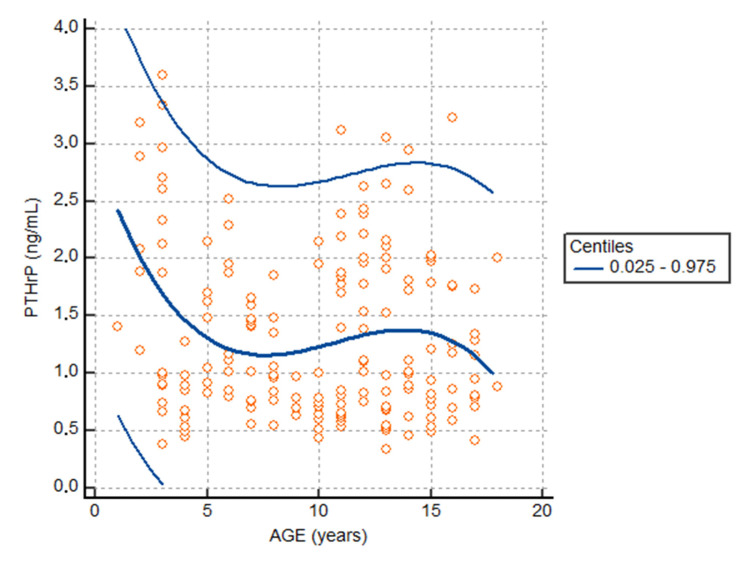
Plot scatter diagram of the measurements versus age. The calculated mean (central line) and centile curves.

**Figure 5:**
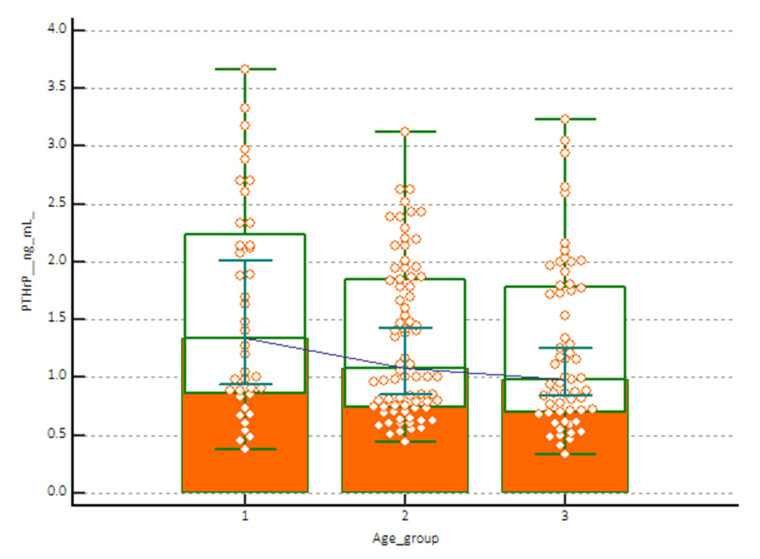
Plot of the scatter diagram of the measurements versus the three groups of subjects evaluated: (1) 1–5 years, (2) 6–12 years and (3) 13–18 years.

The Mann-Whitney test, reported in Table 4, found no significant differences between the median concentrations of PTHrP in the group of female subjects versus male subjects (*p* < 0.05). The Plot of the scatter diagram of concentrations is shown in Figure 4. No significant difference was observed between median concentrations of PTHrP in age group (1–5 years; 6–12 years; 13–18 years) (*p* < 0.05) (Table 4). The scatter diagram of the concentrations in the three age-stratified groups of subjects is shown in Figure 5.

## 5. Discussion and Conclusions

Hypercalcemia is rare in childhood but clinically significant condition. Hypercalcemia may be the product of a malignancy, often mediated by parathyroid hormone-related protein (PTHrP) Although rare, benign tumors can also secrete PTHrP [15,16,17,18,19,20]. An extensive diagnostic workup and management investigation to determine the etiology of this child’s hypercalcemia must be able to highlight the probable overproduction of PTHrP [21,22,27]. Despite these indications, the PTHrP test is performed only by a few laboratories without sufficient standardization in the expression of the results and above all in the availability of specific reference values for the pediatric population. In the literature, there are indications to produce reference values of PTHrP in the healthy pediatric population with a possible evaluation of possible differences related to sex or to differences in age groups [23]. As evidenced by the recent guidelines produced by experts in laboratory medicine, the practice of using normal values obtained from the literature or from other laboratories obtained with methods other than that used in one’s own laboratory can be misleading [28].

It is therefore essential that each clinical laboratory establishes and/or uses appropriate reference ranges for its own methods of PTHrP assay.

The test for PTHrP in use in our laboratory is an EIA kit that doses the peptide fragments of the N-terminal portion (that is, that part of the molecule between (1–86) characterized by good stability and less variability linked to renal function) [11].

To ensure adequate and accurate interpretation of the results of the URL assessment it was essential to select a population of apparently healthy subjects who had suitable homeostasis in calcium/phosphorus metabolism and who were stratified by key covariates: age and sex. The results obtained on the total of pediatric subjects evaluated provided a URL for PTHrP of 2.89 (2.60 to 3.18; 90% CI) pg/mL with an analytical method that has an analytical sensitivity of 0.15 ng/mL. The PTHrP URL we obtained was apparently low and close to the detection limit of the method. This is no different from what has been reported in the literature with other dosing methods. In fact, although the production of PTHrP is widespread in many normal tissues of healthy individuals, its concentration is often low or not detectable with normal analytical methods [14].

PTHrP is a pleiotropic factor with multiple physiological functions in calcium morphogenesis, cell proliferation, differentiation, apoptosis and homeostasis. Therefore, the categories of subjects included in the study could have had hormone levels sensitive to the variations that occur during the neonatal, prepubertal and adrenarchal period. Instead, the data we obtained showed that the variations in PTHrP concentrations and the URL for PTHrP in the age range categories were similar and the differences were not significant. The physiology of PTHrP secretion and the majority of actions on bone development and growth is paracrine in nature, with mainly local control and production [8,9]; this explains the absence of significant variations in serum concentrations in the various age groups evaluated.

As reported in the literature: (i) the HHM syndrome in which PTHrP is produced by the tumor [21,22]; (ii) breastfeeding in which PTHrP is produced in the breast but reaches the circulation [29]; and (iii) fetal life, when PTHrP regulates the transport of calcium from the mother to the fetus [30] are the conditions that present concentrations of PTHrP characterized by elevated levels of circulating hormone as an expression of an endocrine mode of action.

The characterizing element of the study is the evaluation of the reference limit (URL) for the serum PTHrP test in a population of apparently healthy pediatric subjects using the indications for the selection of subjects and for the statistical analysis of the results, reported in the guidelines. CLSI (CLSI C28-A3). The availability of URLs for PTHrP will facilitate the clinical decision-making process, especially in cases of HHM, neoplastic and metabolic pathologies, and will be a stimulus for further future evaluations using different analytical methods. Limitations of the study include an age imbalance across the different age groups with a prevalence of subjects in group (2) (6–12 years). This is to be attributed to the difficulty of obtaining biological material from apparently healthy subjects, especially in the one to five years age group.

## Figures and Tables

**Table 1:** Summary statistics of PTHrP values in all subjects and after stratification by sex (male: PTHRP-M; female PTHrP-F).

Measurements	PTHrP (ng/mL)	PTHrP-M (ng/mL)	PTHrP-F (ng/mL)
**Sample size (n)**	178	98	80
**Highest value**	3.66	3.23	3.66
**Mean**	1.35	1.29	1.43
**95% CI**	1.24 to 1.46	1.14 to 1.43	1.26 to 1.60
**Geometric mean**	1.16	1.11	1.24
**Median**	1.08	1.00	1.20
**Standard deviation**	0.75	0.73	0.78
**2.5–97.5 percentile**	0.45 to 3.12	0.41 to 3.06	0.50 to 3.22
**5–95 percentile**	0.50 to 2.81	0.45 to 2.78	0.53 to 2.83
**Kolmogorov-Smirnov test for Normal distribution**	*p* < 0.0001	*p* < 0.0001	*p* = 0.0016

**Table 2:** An age-related reference interval. A stratification in 9 age groups and a range of PTHrP values at the 2.5–97.5 percentile and 5.0–95.0 percentile, calculated by age group, is reported.

Age Variable	Centiles of PTHrP ng/mL			
(Years)	0.025	0.05	0.95	0.975
2	−1.14	−0.69	3.98	4.43
4	−0.21	0.04	2.67	2.92
6	0.20	0.37	2.23	2.41
8	0.15	0.34	2.24	2.41
10	−0.11	0.11	2.44	2.66
12	−0.38	−0.11	2.65	2.92
14	−0.46	−0.19	2.72	3.00
16	−0.17	0.06	2.47	2.70
18	−0.47	0.44	1.97	2.64

**Table 3:** Summary statistics of PTHrP values in all subjects and stratified by three age groups: 1–5 years; 6–12 years; 13–18 years.

Measurements	PTHrP (1–5 Years) ng/mL	PTHrP (6–12 Years) ng/mL	PTHrP (13–18 Years) ng/mL
**Sample size**	40	78	60
**Lowest value**	0.38	0.44	0.34
**Highest value**	3.66	3.12	3.23
**Arithmetic mean**	1.59	1.31	1.25
**Median**	1.35	1.08	0.99
**Standard deviation**	0.91	0.66	0.72
**Kolmogorov-Smirnov test for normal distribution**	reject Normality (*p* = 0.0044)	reject Normality (*p* < 0.0001)	reject Normality (*p* = 0.0003)

**Table 4:** Comparison of the difference between means in the groups of subjects stratified by sex and age (Mann-Whitney test).

Mann-Whitney Test (Independent Samples)	
Variable	Two-Tailed Probability
PTHrP-M (ng/mL) vs PTHrP-F (ng/mL)	*p* = 0.2305
PTHrP (1–5 years) ng/mL vs PTHrP (6–12 years) ng/mL	*p* = 0.1585
PTHrP (1–5 years) ng/mL vs PTHrP (13–18 years) ng/mL	*p* = 0.0700
PTHrP (6–12 years) ng/mL vs PTHrP (13–18 years) ng/mL	*p* = 0.4653

## Data Availability

All data are available at U.O.C. Clinical Pathology Hospital Policlinico di Bari Italy.
